# Comparison of short and long axis T1 and ECV maps in patients with myocarditis

**DOI:** 10.1186/1532-429X-17-S1-P265

**Published:** 2015-02-03

**Authors:** Sebastian Bohnen, Ulf K Radunski, Gunnar Lund, Lena M Wilmink, Christian Stehning, Gerhard Adam, Stefan Blankenberg, Kai Muellerleile

**Affiliations:** Cardiology, University Heart Center, Hamburg, Germany; Philips Research Europe, Hamburg, Germany; Diagnostic and Interventional Radiology, University Hospital Hamburg Eppendorf, Hamburg, Germany

## Background

T1 mapping and ECV imaging are promising diagnostic tools in patients with suspected myocarditis. However, there are currently no data on the potential influence of slice orientation on the derived T1 and ECV values. Thus, we compared global myocardial T1 and ECV values between slices with short and long axis orientation.

## Methods

This study included 50 patients with clinically suspected myocarditis, who underwent CMR at 1.5 Tesla. T1 quantification was performed on three short (basis, center, apex) and on three long axis slices (2-, 3- and 4-chamber orientation), respectively. The modified Look-Locker inversion-recovery (MOLLI) sequence was used before and 15 minutes after administration of 0.075 mmol/kg Gadolinium-BOPTA. Native T1, post-contrast T1 and extracellular volume (ECV) maps were calculated using a dedicated plug-in written for the OsiriX software. Mean native and post-contrast T1 as well as ECV were obtained from the three short and long axes, respectively.

## Results

There were significantly lower median native myocardial T1 values on long axis slices (1079 (1040-1130.0)) compared to short axis slices (1103 (1069-1140); p=0.0026). However, there were no significant differences in post-contrast myocardial T1 values (median 554 (505-596) vs. 555 (509-601); p=0.2299) or ECV (median 30 (27-33) vs. 29 (26-33); p=0.6063) between short and long axis slices, respectively.

## Conclusions

Slice orientation could be a potential confounding factor for the assessment of native global myocardial T1 values in myocarditis. Possible explanations for this finding include technical issues, such as variations in through-plane motion or partial-volume effects related to slice orientation, but also the heterogeneity of myocardial injury in myocarditis. However, our data indicate that global myocardial ECV values could be less sensitive to slice orientation.

